# Augmented reality navigation systems vs. conventional techniques in acetabular cup positioning: a systematic review and meta-analysis

**DOI:** 10.1016/j.jor.2025.07.005

**Published:** 2025-07-08

**Authors:** Dimitrios Mouselimis, Xanthippi Topalidou, Konstantinos Papadopoulos, Martin Brucker, Gábor Molnár, André R. Zahedi, Christian Lüring

**Affiliations:** a Department of Orthopaedic Surgery, Klinikum Dortmund, Witten/Herdecke University, Dortmund, Germany; b Department of Anesthesiology, Klinikum Dortmund, Witten/Herdecke University, Dortmund, Germany

**Keywords:** Total hip arthroplasty, THA, Augmented reality, AR, Acetabular cup, Complications

## Abstract

**Purpose:**

The aim of this systematic review and meta-analysis was to evaluate the effectiveness of augmented reality (AR) techniques in acetabular cup positioning during total hip arthroplasty (THA).

**Methods:**

The Cochrane Central Register of Controlled Trials, MEDLINE and EMBASE databases were systematically searched until December 12, 2024 according to the PRISMA 2020 guidelines for prospective and retrospective studies comparing AR techniques to conventional ones regarding acetabular cup positioning. Anteversion, inclination angles, blood loss, operation time and postoperative complications comprised the outcomes. Bias assessment was performed with the RoB 2 tool for prospective studies and ROBINS-I for retrospective ones.

**Results:**

In total, 9 studies were included in the systematic review and 8 in the meta-analysis. The target error according to postoperative radiographs or computer scan for both anteversion and inclination angles was significantly more precise in the 346 THAs of the AR group (Z_inclination_ = 3.10, *P*_*inclination*_ = 0.002, Std. mean difference = −0.52 [95 % CI: 0.85 to −0.19] and Z_anteversion_ = 2.44, *P*_*anteversion*_ = 0.01, Std. mean difference = −0.57 [95 % CI: 1.03 to −0.11]) with a significant substantial heterogeneity (I^2^_inclination_ = 79 %, P_inclination_<0.0001 and I^2^_anteversion_ = 89 %, *P*_*anteversion*_ < 0.00001), when compared to the 395 THAs of the conventional group. Regarding blood loss, operation time and postoperative complications, no significant differences were observed. The risk of bias was high among the retrospective studies.

**Conclusion:**

The different types of studies, as well as their individual way of assessing the acetabular cup angles are the main limitations of the meta-analysis. The results provide a strong sign of improved acetabular cup positioning with the help of AR systems, while maintaining a favorable safety profile.

**Prospero registration number:**

CRD42024609350.

## 1 Introduction

Total hip arthroplasty (THA) is recognized as one of the most successful orthopaedic interventions offering patients a rapid recovery and a definite improvement of their quality of life.^1^^,^^2^ Despite its high success rates, potential complications may lead to detrimental consequences for patients’ health. Dislocation of the artificial joint is a common complication conveying a significant risk for periprosthetic fracture and a revising surgery.^3^^,^^4^

The positioning of the cup in the acetabulum is a pivotal point for the postoperative joint function.^5^^,^^6^ Inaccurate implant position has been associated in the literature with an increased risk for dislocation, as well as to limitations in the range of motion and increased wearing of the polyethylene insert.^5^^,^^7^, ^8^, ^9^, ^10^ In recent years, various technological novel implementations of augmented reality (AR) have been developed to overcome the inaccuracy of conventional acetabular cup positioning tools during THA, such as mechanical rods, accelerometers or goniometers.^11^^,^^12^ These AR systems, using a mobile device—typically a smartphone, as seen in the AR Hip platform (Zimmer Biomet Japan, Tokyo, Japan)—allow the surgeon to visualize the anatomy of the pelvis on the device's screen. This assists in accurately positioning the acetabular cup during implantation.^11^ With the help of the AR system the operator has access to both the real intraoperative images and simultaneously to the virtual image provided by the AR system.^11^^,^^12^

AR navigation systems have been developed and introduced into clinical practice since 2009 to provide a solid, radiation-free, and low-cost alternative for older navigation techniques requiring additional diagnostic modalities including computed tomography scans (CT), intraoperative radiographs and robotic assistance.^12^, ^13^, ^14^, ^15^

The aim of this systematic review and meta-analysis was to summarize the latest data in the current literature regarding the comparison of AR navigation techniques to conventional alternatives including mechanical rods and accelerometers in the precision of acetabular cup positioning during THA. Secondarily, the safety of AR instruments was assessed based on surgery time, blood loss and reported number of complications.

## 2 Materials and methods

The systematic review has received the CRD42024609350 number after being registered to the international prospective register of systematic reviews (PROSPERO). The research process including study design, data curation and presentation has been performed in accordance with the Preferred Reporting Items for Systematic Reviews and Meta-Analyses (PRISMA) guidelines.^16^^,^^17^

### 2.1 Eligibility criteria and search strategy

Both retrospective and prospective studies comparing the implementation of AR navigating systems in acetabular cup positioning in THA to conventional methods including techniques with accelerometers (digital or conventional) and mechanical rods have been assessed for inclusion in the systematic review. Inclusion criteria for those studies were primarily the report of inclination and anteversion angles of the implanted acetabular cup and secondarily further clinical outcomes including duration of the surgery and complications including blood loss and fractures. Studies without conventional methods in the control group including accelerometer devices and mechanical rods have been excluded from the review. Only studies written in English were evaluated for study participation.

The Cochrane Central Register of Controlled Trials, MEDLINE and EMBASE databases via the search engines of Cochrane, PubMed, Scopus and Google Scholar were searched by both investigators. The following search terms have been combined with the booleans “AND” and “OR” comprising the search string of the study: (“augmented reality” OR “virtual reality” OR “mixed reality”) AND (“total hip arthroplasty” OR “acetabular cup” OR “acetabular cup positioning” OR “hip arthroplasty” OR “hip”). In the PubMed search engine, the Title/Abstract has been chosen to be searched for the search terms. No other filter or automation tool has been activated during the search process. The Google Scholar search engine was searched only based on titles for any potential articles to be evaluated for addition in the systematic review. A detailed presentation is provided in the supplementary material document. Citation searching of the appropriate articles during the search has also been performed to identify additional studies that may be considered for inclusion in the study. The last search was performed on December 15, 2024.

### 2.2 Study selection process and data extraction

The search results from all databases were initially searched based on the title and abstract. After the first screening the researchers read the whole article and evaluated it according to the inclusion criteria for inclusion in the systematic review. The data extraction was performed only with published data.

The first two authors independently performed the literature search according to the aforementioned criteria. In cases of disagreement, a consensus was reached in consultation with the other authors. The following parameters were collected:

• Study design: (1) Navigation system with AR technology and conventional alternative, (2) angle evaluation method with radiographs or CT scan, (3) materials used, (4) surgical technique, (5) number of operators
• Results: (1) Anteversion and inclination angles, (2) clinical outcomes and complications, (3) basic demographic characteristics including age and sex

### 2.3 Data analyses

The meta-analysis was performed with the Review Manager (RevMan, Version 5.4. The Cochrane Collaboration, 2020.). The angles of anteversion and inclination and the mean differences of them presented as mean ± SD were included in the forest plots. Data presented as median and IQR were excluded from the meta-analysis. The same rule was followed also for the remaining parameters including blood loss and surgery time. Due to the different type of studies included (retro- and prospective) the random effect model for the standardized mean difference was utilized in the analysis of pool estimates, due to the significant variability between the different systems used in each study. A sensitivity analysis was performed according to studies’ characteristics to assess any significant change in the results. Moreover, a one-by-one exclusion-inclusion analysis has been performed in order to evaluate any significant impact on the pooled outcomes. Heterogeneity has been assessed according to the Cochrane Handbook for systematic reviews and meta-analyses.^18^

### 2.4 Inclination and anteversion angles

In the meta-analysis, the difference between the prespecified target inclination and anteversion angles and the radiographic angles measured postoperatively has been chosen if available. In the case that only the navigation error has been reported, it had to be included in the result. The navigation error is expressed as the mean-difference between the intraoperative measured angle and the radiographically measured postoperative angles. Separate sensitivity analyses were performed to show any significant differences. A comparison regarding the navigation error was not feasible in all studies, such as in the case that in the control group mechanical rods were used as navigation. If data were available from both radiographs and computer tomography (CT), the CT results has been used in the meta-analysis.

### 2.5 Risk of bias

The risk of bias assessment was separately performed by the first two authors. In cases of disagreement a consensus with the help of the remaining team was conducted. The Risk Of Bias In Non-randomized Studies - of Interventions (ROBINS-I) was used for the non-randomized studies,^19^ whereas the Version 2 of the Cochrane Risk-of-Bias tool for Randomized trials (RoB 2) was chosen for the randomized studies.^20^ Due to the preliminary level of development of the investigated AR systems there was performed no thorough assessment of the quality of evidence.

## 3 Results

**Fig. 1:**
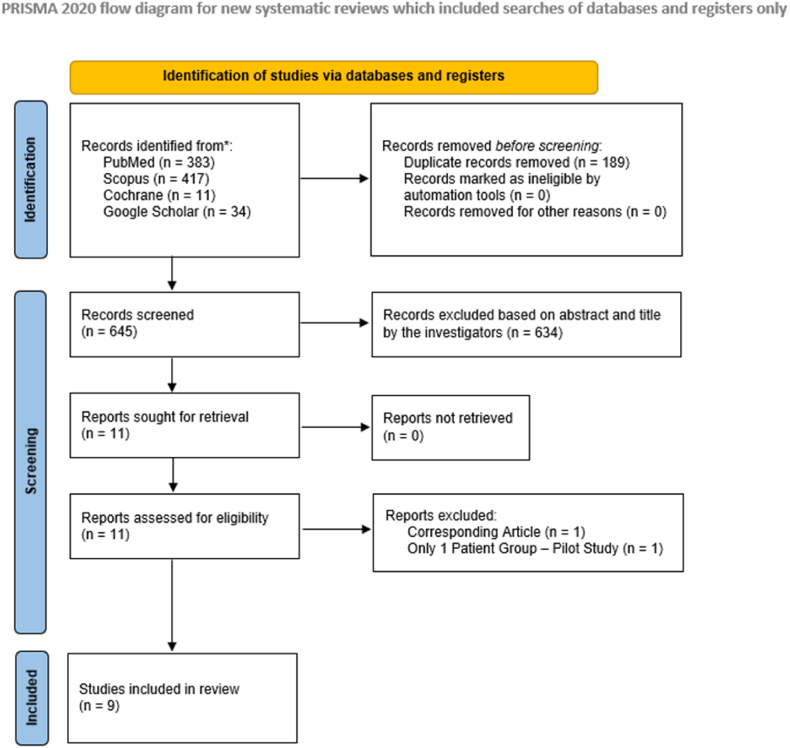
The PRISMA 2020 Flowchart.

The complete workflow of the study selection is presented in the PRISMA flow-diagram (Fig. 1). In total, 9 studies have been included in the systematic review.^12^^,^^21^, ^22^, ^23^, ^24^, ^25^, ^26^, ^27^, ^28^ These are presented in Table 1. In total, 780 patients underwent 813 THAs (Table 1). Their demographic characteristics including Age, Sex and BMI are presented in Table 1. The study from Tanino et al. 2023^22^ could not be included in the meta-analysis due to reporting the data as median and IQR.

**Table 1:** The included studies in the systematic review comparing augmented reality navigation tools vs. conventional techniques in acetabular cup positioning.

Study	Type of study and land	System tested	Patients	Outcomes	Surgical approach and types of implants and complications	Notes
Ogawa H et al. 2020^28^	-Prospective, 2 arm randomized controlled trial with 1:1 treatment distribution (assessor-blind) -Japan	- AR portable navigation system recognizing inserted pins vs. conventional free-hand and vs goniometer based on pre and postoperative CT scan and radiographs. - The postoperative radiograph was performed direct postoperatively, whereas the CT 3 months later	- AR group (N = 22): 86 % women, mean age of 65 ± 11 years, BMI of 22.9 ± 3.8 kg/m^2^ (range 16.6–32.7) - Conventional group (N = 19): 90 % women and mean age of 67 ± 12 years, BMI of 22.6 ± 4.4 kg/m^2^ (range 16.6–38.0)	Mean difference of target angle AR system vs. conventional group based on postoperative CT and radiographs: - Inclination based on CT: target angle 1.9° ± 1.3° (95 % CI: 1.3–2.5) vs. 3.4° ± 2.6° (95 % CI: 2.1–4.6), ***P* = .02** and 95 % CI: 0.2 to 2.8 - Inclination based on radiographs: 2.3° ± 1.4° (95 % CI: 1.7–2.9) vs. 3.9° ± 2.4° (95 % CI: 2.8–5.1) ***P* = .009** and 95 % CI: 0.4 to 2.8 - Anteversion based on CT: 2.8° ± 2.2° (95 % CI: 1.9–3.8) vs 3.8° ± 3.0° (95 % CI: 2.4–5.3), P = .22 and 95 % CI: 0.6 to 2.6 - Anteversion based on radiographs: 2.3° ± 1.8° (95 % CI: 1.5–3.1) vs. 2.9° ± 2.2° (95 % CI: 1.8–4.0), P = .34 and 95 % CI: 0.6 to 1.9 Mean difference of navigation error AR system vs the goniometer based on postoperative CT and radiographs: - Inclination error based on CT: target angle 2.0° ± 1.5° (95 % CI: 1.5–2.5) vs. 3.1° ± 2.3° (95 % CI: 2.4–3.9), ***P* = .009** and 95 % CI: 0.3 to 2.0 - Inclination error based on radiographs: 2.9° ± 2.1° (95 % CI: 2.2–3.5) vs. 3.5° ± 2.2°/2.3° (95 % CI: 2.8–4.2) *P* = .19 and 95 % CI: 0.3 to 1.6 - Anteversion error based on CT: 2.9° ± 2.0° (95 % CI: 1.3–3.5) vs 7.5° ± 3.6° (95 % CI: 6.4–8.6), ***P* = .001** and 95 % CI: 3.3 to 5.9 - Anteversion error based on radiographs: 2.5° ± 2.1° (95 % CI: 1.8–3.2) vs. 5.3° ± 3.2° (95 % CI: 4.3–6.4), ***P* = .001** and 95 % CI: 0.6 to 1.9 - Operation time AR vs conventional group: 43 ± 7 vs. 44 ± 12 min, *P* = .8 - Intraoperative blood loss AR vs conventional group: 136 ± 44 vs. 147 ± 69 min P = .52 - No complications were recorded in both groups	- Modified Watson-Jones approach in the lateral decubitus position - No data for the implanted material - No important complications including fractures or infections for both groups	- Single surgeon - Software for the CT measurements: ZedHip; LEXI, Tokyo, Japan - Software for the x-ray measurements: Kyocera Medical, Osaka, Japan - Two blinded assessors performed the image analysis - Anteversion target angle 40° and Inclination target angle 15° or 20° based on patients' characteristics according to the functional pelvic plane - All patients received general anesthesia
Tsukada et al. 2022^27^	- Retrospective, 2 arm - Japan	- AR Hip (Zimmer Biomet Japan, Tokyo, Japan) vs. an accelerometer-based navigation system (HipAlign system) based on postoperative X-rays	- AR group (N = 45): 81 % women, mean age of 66 ± 9 years, BMI of 24.7 ± 3.9 kg/m^2^ - Accelerometer group (N = 42): 84 % women and mean age of 62 ± 11 years, BMI of 25.0 ± 4.2 kg/m^2^	Mean difference of navigation error AR system vs. accelerometer group based on postoperative radiographs: - Inclination: 2.5° ± 1.7° vs 4.6° ± 3.1° (95 % CI 1.1°–3.2°), ***P* < .0001** - Anteversion: 2.1° ± 1.8° vs 6.4° ± 4.2° (95 % CI: 3.0°–5.7°), ***P* < .0001** - Operation time AR vs conventional group: 48 ± 12 vs. 49 ± 9 min, *P* = .47 - Intraoperative blood loss AR vs conventional group: 199 ± 95 vs. 203 ± 97 min *P* = .84 - In the HipAlign group one patient suffered a periprosthetic fracture. No other complications were recorded in both groups.	- Modified Watson-Jones approach in the lateral decubitus position - Cementless acetabular cup in all patients, 59 hips with G7 Zimmer Biomet, Warsaw, IN and 28 hips with Trident, Stryker, Mahwah, NJ - Uncemented femoral stem: Avenir (Zimmer-Biomet) in 34 hips, Accolade II (Stryker) in 17 hips, Optimys (Mathys, Bettlach, Switzerland) in 14 hips, Corail (DePuy Synthes, Raynham, MA) in 8 hips, Entrada (Japan MDM, Tokyo, Japan) in 8 hips, SL-Plus MIA HA (Smith & Nephew, London, UK) in 2 hips, Mainstay (Kyocera, Osaka, Japan) in 1 hip, and Wagner Cone (Zimmer Biomet) in 1 hip. - Cemented stem: Exeter (Stryker) in 2 hips.	− 3 experienced surgeons - Portable navigations systems: HipAlign (OrthAlign, Aliso Viejo, CA) and AR-Hip (Zimmer Biomet Japan, Tokyo, Japan) - Software for the x-ray measurements: Kyocera Medical, Osaka, Japan - Inclination target angle 40° and anteversion target angle 15°–25° based on patients' characteristics - Two Assessors blinded to intervention performed the image analysis - No data for anesthesia
Fujita et al. 2023^26^	- Retrospective, 2 arm study - Japan	AR Hip (Zimmer Biomet Japan, Tokyo, Japan) vs. an accelerometer-based navigation system (HipAlign system) based on postoperative CT scans	− 33 patients in each group undergoing 35 THAs - AR group (N = 33): 77 % women, mean age of 64.4 ± 14.7 years, BMI of 23.5 ± 3.4 kg/m^2^ - Accelerometer group (N = 33): 83 % women and mean age of 67.1 ± 10.4 years, BMI of 22.9 ± 3.4 kg/m^2^	Mean difference of navigation error AR system (N_hips_ = 35) vs. accelerometer group (N_hips_ = 35) based on postoperative CT scans: - Inclination: 2.7° ± 1.8° vs 2.8° ± 2.6°, *P* < .879 - Anteversion: 2.6° ± 2.2° vs 2.5° ± 2.0°, *P* < .746 - Duration of surgery AR vs conventional group: 78.4 ± 5.9 vs. 76.5 ± 2.7 min, *P*-value not available - No data regarding blood loss - No complications were recorded for both groups - The absolute measurement error (≥3°) of cup inclination and patients' BMI in the Hip Align group and not in the AR-Hip group were significantly associated: Odds ratio 1.350, 95 % CI: 1.035–1.760; ***P* = .027**	- Spine position - AR group: 33 patients (35 hips) with G7 Osseo Ti cup and Polar stem (Smith & Nephew, Baar, Switzerland) - Hip Align system: 33 patients (35 hips) with G7 Osseo Ti cup and Fitmore or Taperlock Microplasty stem (Zimmer-Biomet, Warsaw, IN)	- One experienced surgeon - Software for the CT measurements Zed hip; Lexi - No complications - Inclination angle was fixed at 40° and the anteversion angle based on a preoperative 3D template according to Widmer's combined anteversion theory - The assessors were not blinded to the intervention (no information)
Kurosaka et al. 2023^25^	- Prospective, 2-arm, parallel-group, randomized controlled trial, (blinded assessor) - Japan	AR Hip (Zimmer Biomet Japan, Tokyo, Japan) vs. an accelerometer-based navigation system (HipAlign system) based on postoperative X-rays	- AR group (N = 62): 81 % women, mean age of 67 ± 10 years, BMI of 24 ± 4 kg/m^2^ - Accelerometer group (N = 64): 86 % women and mean age of 69 ± 10 years, BMI of 25 ± 5 kg/m^2^	Mean difference of navigation error AR system vs. accelerometer group based on postoperative radiographs: - Inclination: 3° ± 2° vs 3° ± 2° (95 % CI: 1.2°–0.3°), *P* < .22 - Anteversion: 2° ± 2° vs 5° ± 4° (95 % CI: 4.2° to −2.0), ***P* < .001** - Duration of surgery AR vs conventional group: 45 ± 9 vs. 48 ± 10 min, P = .1 - Intraoperative blood loss AR vs conventional group: 178 ± 84 vs. 183 ± 102 min *P*-value not available - Complications in the AR group: 1 surgical site infection, 1 inraoperative fracture, 1 distal deep vein thrombosis and 1 intraoperative loosening of navigation pins, and in the HipAlign group: 1 intraoperative fracture and 1 loosening of the navigation pins	- Modified Watson-Jones approach - General Anesthesia - cementless acetabular cups in all patients (G7, Zimmer Biomet) − 119 stems were cementles whilst 7 were cemented	− 3 surgeons - The software for the X-ray analysis was the Two-Dimensional Template computer software Kyocera Medical, Osaka, Japan - Inclination target angle 40° and anteversion target angle 15°–25° based on patients' characteristics - Few complications in both groups - Two blinded assessors performed the image analysis - All patients underwent general anesthesia
Tanino et al. 2023^22^	- Prospective, 2-arm, parallel-group, randomized controlled trial, (no report for a blinded assessor) - Japan	AR-Hip (Zimmer Biomet Japan, Tokyo, Japan) vs. Conventional mechanical guide based on postoperative CT scans	- AR group (N = 36): 83 % women, mean age of 65 ± 11 years, estimated BMI of 26.1 ± 6.34 kg/m^2^ - Conventional group (N = 36): 83 % women and mean age of 69 ± 10 years, estimated BMI of 24.6 ± 7.1 kg/m^2^	Median difference of target angle AR system vs. conventional group based on postoperative radiographs: - Inclination: 1° (IQR 0°–4.0°) vs. 5°, (IQR 3.0°–7.5°), difference of medians 4°, ***P* < .001** - Anteversion: 2° (IQR 1.9°–3.7°) vs. 5°, (IQR 3.2°–9.7°), difference of medians 2°, ***P* < .001** - Duration of surgery AR vs conventional group in this case as median and IQR: 58 (49–72) vs. 68 (49–69) minutes, *P* = .1 - No data for blood loss - No complications in both groups	- Standard posterior approach with repair of the posterior soft tissue in a lateral decubitus position - All patients received a cementless, hemispherical acetabular component (Continuum®, Zimmer Biomet) - Most patients received cemented CMK Original Concept stems (Zimmer Biomet) − 7 hips received a modular cementless stems (SROM ®, DePuy) were used in seven hips. - All patients received 32-mm ceramic heads.	− 2 senior surgeons - Software for CT analysis: ViewR, YOKOGAWA measured by one observer - Anteversion target angle 40° and Inclination target angle 20° - Only medians reported - Only one assessor for the images (no information for being blinded to the intervention) - No information for administered anesthesia
Tanaka et al. 2024^24^	- Retrospective 2 arm study - Japan	AR-Hip (Zimmer Biomet Japan, Tokyo, Japan) vs. second generation accelerometer based portable navigation system (Naviswiss; Naviswiss AG, Brugg, Switzerland) based on postoperative CT scans	- AR group (N = 69): 75 % women, mean age of 66 ± 12 years with 87 cementless stems, BMI of 24.7 ± 4.8 kg/m^2^ - Conventional group (N = 89): 82 % women and mean age of 67 ± 10 years with 93 % cementless stems, BMI of 23.2 ± 3.5 kg/m^2^	Mean difference of target angle AR system vs. accelerometer group based on postoperative CT: - Inclination: 3.5° ± 3.1°vs 3.5° ± 3.0°, *P* = .751 - Anteversion: 4.5° ± 4.0° vs 4.1° ± 3.1°, *P* = .719 Mean difference of navigation error AR system vs. accelerometer group based on postoperative CT: - Inclination: 3.0° ± 3.2°vs 2.9° ± 2.7°, *P* = .862 - Anteversion: 4.3° ± 4.1° vs 4.3° ± 3.5°, *P* = .938 - Duration of surgery AR vs conventional group: 95 ± 21 vs. 91 ± 23 min, *P* = .255 - Intraoperative blood loss AR vs conventional group: 303 ± 214 vs. 333 ± 220 ml, *P* = .387 - Complications in the AR group: 3 Dislocations, 2 intraoperative fractures, 2 infections of the surgical site and 1 Nerve palsy, and in the Naviswiss (conventional) group 2 intraoperative fractures and 2 surgical site infections	- lateral decubitus position with a standard posterior approach - AR group: G7 (Zimmer Biomet, Warsaw, IN, USA) acetabular cup, 42 patients received a Taperloc Complete Microplasty stem, 16 an Avenir stem, 5 an CMK stem, 4 a CPT stem and 2 a Wagner Cone stem - Naviswiss group: (Kyocera, Osaka, Japan) acetabular cup was used, 83 patients received an Initia stem, 2 patients a SC stem 3 patients an SN–C stem in three patients, and 1 patienat a 7 stem.	− 4 surgeons − 24 patients in each group underwent lumbar anesthesia, whilst the remaining general anesthesia - Anteversion target angle 40° and Inclination target angle 20° - 3D CT template software ZedHip; Lexi, Tokyo, Japan - The assessors were not blinded to the intervention (no information)
Shimizu et al. 2024^12^	- Retrospective 2 arm study - Japan	AR-Hip (Zimmer Biomet Japan, Tokyo, Japan) vs. conventional technique with mechanical based on postoperative CT scans	- In total, 45 hips were in included in both groups - AR group (N = 43): 77 % women, mean age of 66.7 years with a range of 36–93, BMI of 24.1 with a range of 16.8–32 kg/m^2^ - Conventional group (N = 45): 76 % women and mean age of 62.9 years with a range of 46–87, BMI of 24.6 with a range of 17.8–46.2 kg/m^2^	- Mean difference AR system (N_hips_ = 45) vs. conventional group (N_hips_ = 45) based on postoperative CT: - Inclination: 2.6° ± 2.11°vs 4.61° ± 3.28°, ***P* = .0036** - Anteversion: 3.57° ± 3.36° vs 3.87° ± 2.97°, *P* = .4732 - The mean difference in AR group was based on the intraoperative displayed angle, whilst in the conventional group on the predefined target angles - No outcomes regarding operation time and blood loss - No data regarding complications	- Lateral OCM approach - All cups were G7OsseoTi® (Zimmer-Biomet) - No data for implanted stems	- No data regarding anesthesia - Anteversion target angle 40° and Inclination target angle 20° - CT template software ZedHip; Lexi, Tokyo, Japan - No data for operators and assesors of the postoperative images
Kimura et al. 2024^23^	- Retrospective 2 arm pilot study - Japan	Novel AR pinless system vs. conventional technique with mechanical rods based on postoperative radiographs	− 44 patients received a unilateral THA, whilst 14 patients received a bilateral THA in a single operation. In total 72 THAs were included in the study - Pin-less AR group (N = 27): 85 % women, mean age of 60.4 ± 8.6 years, BMI of 23.6 ± 5.6 kg/m^2^ - Conventional group (N = 31): 87 % women and mean age of 63.4 ± 9.6 years, BMI of 23.2 ± 4.9 kg/m^2^	- Mean difference of target error between the pin-less AR system (N_hips_ = 31) vs. conventional group (N_hips_ = 38) based on postoperative radiographs: - Inclination: 1.9° ± 1.5° vs 4.2° ± 2.7°, ***P* < .001** - Anteversion: 2.3° ± 1.5° vs 3.1° ± 2.1°, ***P* < .001** - Mean difference of navigation error between the pin-less AR system (N_hips_ = 31) vs. conventional group (N_hips_ = 38) based on postoperative radiographs: - Inclination: 2.2° ± 1.4° vs 4.2° ± 2.7°, *P*-value not available - Anteversion: 2.4° ± 1.5° vs 3.1° ± 2.1°, *P*-value not available - Operation time AR vs conventional group per THA: vs. 41.3 ± 10.5 vs 36.4 ± 12 min, ***P* = .082** - Intraoperative blood loss AR vs conventional group per THA: 324.5 ± 156.4 vs. 280.6 ± 150.7 ml, *P* = .25 - No patients had significant complications	- In the AR group patients received a G7® Acetabular System (Zimmer Biomet, Warsaw, IN, USA) cup, while the conventional group received an Anasta Cup (Teijin Nakashima Medical Co. Ltd., Okayama, Japan) or Pinnacle Acetabular Cup (Depuy, Warsaw, IN, USA) - Different type of stems were utilized in the study	- Performed by 2 surgeons - No information regarding anesthesia - The target angles were 40° inclination and 20° anteversion - The software for analysis of the radiographs was the Hip Scouter (Zimmer Biomet G.K., Tokyo, Japan) - No data regarding assessors of the images
Naito et al. 2024^21^	- Retrospective 2 arm pilot study - Japan	CT-based AR system (Holonavi Medical Technology Inc., Ichinomiya, Japan) vs. conventional technique with mechanical rods based on postoperative CT scans	− 33 patients underwent 37 primary cementless THAs in the AR group, whereas 51 patients underwent 63 THAs in the conventional group - CT-based AR group (N = 33): 84,9 % women, mean age of 66 with a range of 37–79 years, BMI of 25 with a range of 18–46 kg/m^2^ - Conventional group (N = 51): 87 % women and mean age of 63 with a range of 45–86 years, BMI of 24 with a range of 15–37 kg/m^2^	- Mean difference of the target angles between the pin-less AR system (N_hips_ = 37) vs. conventional group (N_hips_ = 63) based on postoperative radiographs: - Inclination: 2.9° ± 1.8° vs 8.4° ± 7°, ***P* < .001** - Anteversion: 2.9° ± 2.2° vs 11.6° ± 6.6°, ***P* < .001** - Mean difference of the navigation error between the pin-less AR system (N_hips_ = 37) vs. conventional group (N_hips_ = 63) based on postoperative radiographs: - Inclination: 2.9° ± 2.1° vs 8.4° ± 7°, *P*-value not available - Anteversion: 3.3° ± 2.4° vs 11.6° ± 6.6°, *P*-value not available - Duration of surgery AR vs conventional group per THA: 130 ± 31 vs. 112 ± 30 min, ***P* < .005** - No data for intraoperative blood loss - No data regarding complications	- Posterior surgical approach in the lateral decubitus position - In the AR group 31 THA were performed with a SQRUM TT SHELL (Kyocera, Osaka, Japan) and 6 hips with a G7 (Zimmer Biomet, Warsaw, IN, USA). In the conventional group in 33 hips a Regenerex Ringloc Acetabular Component was used, in 22 a Continuum Acetabular Shell, in 7 a G7 PPS Finned BoneMaster Acetabular Shell and in 1 hip a Trilogy Acetabular Shell (all components were from Zimmer Inc., Warsaw, IN).	- All operations were performed by a single surgeon - No information regarding anesthesia - The target angles were 40° inclination and 15° anteversion for the AR group and 40° and 20° (radiographically 42° and 15°) for the conventional group respectively - 3D CT template software ZedHip; Lexi, Tokyo, Japan - The same independent observer evaluated the images

Navigation error – mean difference between the postoperatively measured angle and the intraoperatively shown on the display of the AR system angle; AR – augmented reality; BMI – body mass index; CT – computed tomography; SD – standard deviation; IQR – interquartile range; OCM – Orthopädische Chirurgie München.

Only 2 studies were prospective randomized controlled trials.^25^^,^^28^ In total, 5 studies^22^^,^^24^, ^25^, ^26^, ^27^ have used the AR Hip system (Zimmer Biomet Japan, Tokyo, Japan), whereas 3 of them^25^, ^26^, ^27^ compared it to the Hip Align system. A complete panorama of the studies, their design and outcomes is presented in Table 1.

### 3.1 Anteversion and inclination

In total, the pooled mean difference between the target angle and postoperative angle was statistically significant for both anteversion and inclination. In total, 346 hips were included in the AR-group and 395 in the conventional group. A compromise regarding the target angle has been done in 4 studies reporting the navigation error.^12^^,^^25^, ^26^, ^27^ Regarding inclination, the Z = 3.10 for a *P* = .002 with a statistically significant substantial heterogeneity (I^2^ = 79 % *P* < .0001). Regarding the navigation error of inclination, 309 THAs implanted with the help of AR were compared with 332 conventional implanted THAs. The results were similar to those for the target error (Z = 2.48, *P* = .01, I^2^ = 75 % *P* = .0006).

**Fig. 2:**
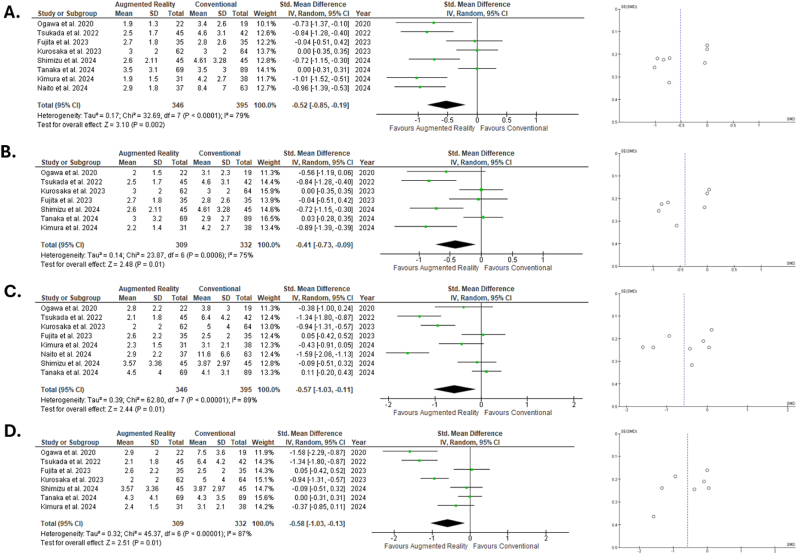
Forest and funnel plots of inclination and anteversion angles A. Standardized mean difference of target error in inclination angle B. Standardized mean difference of navigation error inclination angle C. Standardized mean difference of target error in anteversion angle D. Standardized mean difference of navigation error in anteversion angle.

The comparison of the two groups for the target error in anteversion angle revealed a statistically significant Z = 2.44 (*P* = .01) with statistically significant substantial heterogeneity (I^2^ = 89 % *P* < .00001). Similar was the result for the navigation error in anteversion angle (Z = 2.51, *P* = .01, I^2^ = 87 % *P* < .00001). All forest plots accompanied by their funnel plots are presented in Fig. 2(A–D). A thorough sensitivity analysis revealed no significant alterations of the results.

### 3.2 Blood loss and operative time

**Fig. 3:**
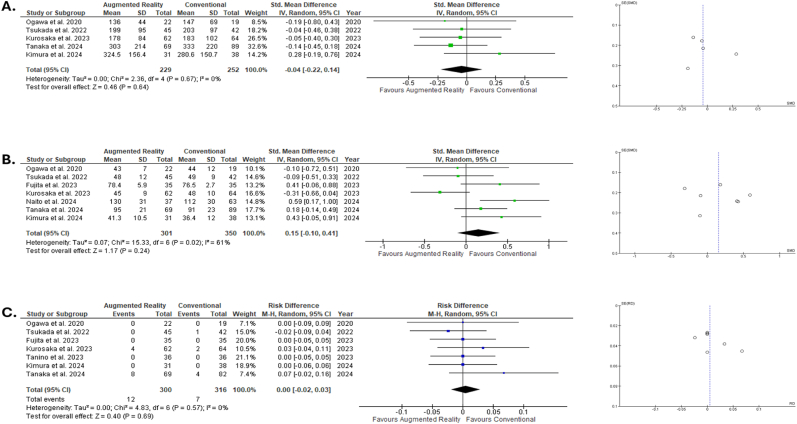
Forest and funnel plots regarding blood loss (A), operation time (B) and postoperative complications (C).

The comparison regarding the blood loss between the 229 hips in the AR group to the 252 hips in the conventional group revealed no statistically significant difference (Z = 0.46, *P* = .64), while the heterogeneity between the included studies remained to 0 % (*P* = .67). See Fig. 3A.

The operation time in the AR group including 301 hips did not differ statistically significantly compared to the conventional group, which included 350 patients (Z = 1.17, *P* = .24). The heterogeneity in this comparison was statistically significant substantial (I^2^ = 61 %, *P* = .02). See Fig. 3B.

### 3.3 Postoperative complications

In a total number of 300 THA in the AR group and 316 in the conventional group only 19 significant adverse events were recorded. The comparison of the risk difference between the two groups was characterized by a non-statistically significant Z = 0.40 (*P* = .69) with no heterogeneity (I^2^ = 0, *P* = .57) and is presented in Fig. 3C.

### 3.4 Risk of bias assessment

**Fig. 4:**
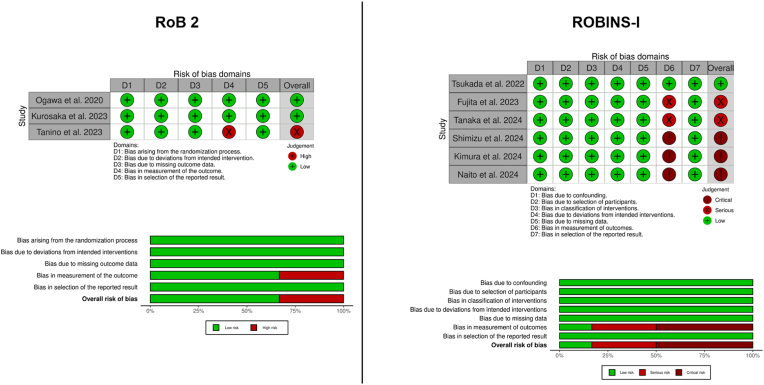
Risk of Bias in the included studies.

Only 1 prospective study has been characterized having a high risk of bias due to a not blinded assessor.^22^ Regarding the retrospective studies a significant risk of bias has been yielded due to not blinded assessors or due to no correction of systematic errors in the radiographs assessment. A detailed presentation is provided in Fig. 4.

## 4 Discussion

Our study provides the latest data on the use of AR navigation systems in acetabular cup positioning. In total, 9 studies have been yielded by the systematic research and have been included in the systematic review.^12^^,^^21^, ^22^, ^23^, ^24^, ^25^, ^26^, ^27^, ^28^ One of them was excluded in the meta-analysis as the reported data were in the form median and IQR and were therefore not suitable for inclusion in the meta-analysis.^22^ The most commonly utilized AR system was the AR Hip system (Zimmer Biomet Japan, Tokyo, Japan).^22^^,^^24^, ^25^, ^26^, ^27^ In only one study a pin-less AR system was used,^23^ whilst in all others an additional implantation of bony landmarks has been a prerequisite for the AR system's proper function. The precision of the acetabular cup positioning with the help of AR systems has been shown to be greater than conventional techniques. The reported navigation error remained significantly lower in the AR group regarding both inclination and anteversion angles. The safety of the AR implementation has also been proven based on the non-statistically significant results for blood loss, operation time and postoperative complications.

Our meta-analysis presents a significant addition of newly published studies since the last published meta-analysis.^29^ Although the present study was published just one year later, it included nearly twice as many patients (780 vs. 396), who underwent a THA compared to the latest meta-analysis. The results confirm those from a year ago, while heterogeneity remained at high levels. This highlights how rapidly the field of augmented reality is advancing and suggests that it will likely play a significant role in the future. No assessment of the evidence was performed due to the preliminary status of the presented AR systems.

The importance of the precise positioning of the acetabular cup has been underlined by multiple studies in the past.^9^^,^^30^^,^^31^ The widely adopted method of implanting the cup in the Lewinnek's safe zone does not ensure always a low luxation rate according to systematic research of the literature.^5^ The results of a cohort study incorporating more than 9000 THAs questioned the safety zone regarding the dislocation risk, proposing further investigation in the field focusing on a more individual acetabular cup placement.^32^ The spinopelvic tilt, is a significant confounding factor that has to be considered in the implantation of the acetabular cup.^33^ The need for precise implantation of the acetabular cup based on individual patient characteristics renders the use of new technology pivotal for the future.

Robotic assisted arms have been proven to offer a clinically safe and precise implantation of the acetabular cup.^34^, ^35^, ^36^, ^37^, ^38^ In a study utilizing the Mako system (Stryker, Kalamazoo, MI, US) to assess the precision of the acetabular cup positioning in dysplastic hips, The results showed a significant higher precision within 5° of the target of the robotic arm compared to CT-assisted navigation (P = .0049) and manual technique (P < .0001).^39^ These results came to enhance the assumptions of prior studies pointing out the accuracy of the robotic arms in acetabular cup positioning.^40^

The use of the AR navigation systems seems to be the only worth considering alternative to robotic arm assisting systems in THAs in individual positioning of the acetabular cup. According to the results of our study, the AR navigation systems are a safe alternative that offers precise implantation rates when compared to conventional techniques. Further studies analysing the cost-effectiveness of both implementations, as well as a direct comparison of both regarding precision and safety will provide important details for the daily clinical practise.

Although the results of our meta-analysis are promising in favour of the AR navigation systems, there are some important aspects that remain to be clarified. Most of the studies included in the meta-analysis are retrospective, while there are many issues raised from the bias assessment regarding postoperative assessment of the acetabular cup position. Moreover, not all the studies reported target angles introducing in this way a potential bias in the results. However, the subgroup analysis for studies reporting only the navigation error presented the same superiority of the AR systems. In all comparisons the heterogeneity remained significantly high, which is attributed to the different study designs, the different surgical centres and utilized AR and radiographic systems.

## CRediT authorship contribution statement

**Dimitrios Mouselimis:** Conceptualization, Methodology, Project administration, Data curation, Formal analysis, Investigation, Supervision, Statistical Analysis, Writing – original draft, Visualization. **Xanthippi Topalidou:** Systematic search strategy, Statistical Analysis, Data extraction, Risk of bias assessment, Validation, Writing – review & editing. **Konstantinos Papadopoulos:** Data extraction, Data interpretation, Writing – review & editing. **Martin Brucker:** Methodology, Critical revision, Writing – review & editing. **Gábor Molnár:** Methodology, Critical revision, Writing – review & editing. **André R. Zahedi:** Methodology, Critical revision, Writing – review & editing. **Christian Lüring:** Conceptualization, Supervision, Methodological oversight, Writing – review & editing.

## Human ethics and consent to participate declarations

Not applicable.

## Ethical statement

None to declare.

## Patient's consentd

Not applicable.

## Declaration of generative AI in scientific writing

None.

## Funding statement

None to declare.

## Declaration of competing interest

None.
